# Fibrolamellar Carcinoma: A Rare Liver Neoplasm

**DOI:** 10.7759/cureus.59006

**Published:** 2024-04-25

**Authors:** Elily D Apumayta, Aaron Kahlam, Eloy F Ruiz

**Affiliations:** 1 Department of Abdominal Surgery, Instituto Nacional de Enfermedades Neoplasicas, Lima, PER; 2 Department of Internal Medicine, Rutgers New Jersey Medical School, Newark, NJ, USA

**Keywords:** hepatocellular, carcinoma, liver cancer, peru, fibrolamellar carcinoma

## Abstract

Fibrolamellar carcinoma is a rare liver tumor, with most cases arising in people younger than 40 years of age. We present a case series of five patients with histological confirmation of fibrolamellar carcinoma who had liver resection as the primary treatment. The median age of diagnosis was 24 years with nonspecific clinical manifestations in otherwise healthy patients. Alpha-fetoprotein levels were widely variable. Patients had classical imaging, macroscopic, and microscopic findings. Most of our patients underwent a hemihepatectomy and 60% recurred after the first year.

## Introduction

Fibrolamellar carcinoma (FLC), initially described in 1956 by Edmondson as a subtype of hepatocellular carcinoma (HCC), is now considered a distinct entity with its specific gene expression profile [1,2]. This rare neoplasm accounts for less than 1% of all primary liver tumors [1,3]. It has a bimodal distribution with two peaks, one between ages 15 and 19 years old, and another between ages 70 and 74 years old [4]. Most cases arise in people younger than 40 years of age [4,5] and there is no apparent gender predilection [1]. Patients have no underlying liver disease and can be asymptomatic or may have non-specific symptoms such as abdominal distention or palpable mass [1,3].

Fibrolamellar carcinoma has several hallmark findings that are useful for diagnosing the disease. The classical radiological findings include a heterogeneous and well-circumscribed mass in a non-cirrhotic liver [6]. Grossly, FLC is a yellow to light gray, large, solitary, and unencapsulated tumor with a central scar [1,2]. Histologically, the tumor is made up of large polygonal cells containing abundant eosinophilic cytoplasm, large vesiculated nuclei, and large nucleoli, with tumor cells that are embedded in lamellar bands of fibrosis [1,2].

The diagnosis of FLC is based on the clinical presentation, imaging studies, and histopathology. Complete surgical resection with intraoperative exploration of locoregional disease improves survival and is the gold standard for treatment [2,5]. Unfortunately, there is no effective adjuvant or neoadjuvant systemic therapy for FLC [2]. This study aims to describe the disease findings and outcomes from a series of five cases with FLC diagnosed between 2010 and 2021 at the National Institute of Neoplastic Diseases in Lima, Peru.

## Case presentation

Case 1

**Figure 1:**
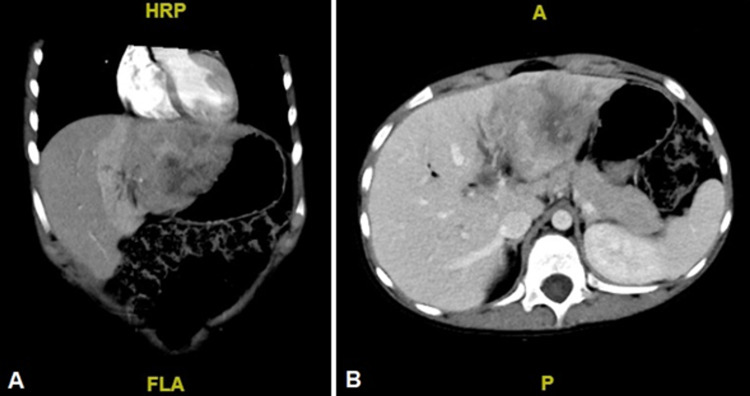
Contrast-enhanced abdominal CT, venous phase. Heterogeneous liver lesion in Segments II and III, with hypodense areas in relation to necrosis and apparent satellite nodules in Segment II. A: Coronal view. B: Axial view

A nine-year-old female presented with a three-month history of vague abdominal pain and palpable hepatomegaly. A blood workup revealed elevated gamma-glutamyltranspeptidase (GGT, 269 IU/L) and alkaline phosphatase (ALP, 404 IU/L) with normal bilirubin level and transaminases. The alpha-fetoprotein (AFP) was 2639 ng/mL on initial assessment. Serology for viral hepatitis A, B, and C were negative. The abdominal computed tomography (CT) scan showed a heterogeneous lesion in liver Segments II and III, associated with satellite lesions in Segment II (Figure 1). The patient underwent left hemihepatectomy plus segmentectomy of Segment I and Kehr drain placement without complications. A 6.5 cm tumor was resected with clean surgical margins and no evidence of lymphovascular invasion (LVI), perineural invasion (PNI), or regional lymph node involvement. Moderately differentiated FLC was later confirmed on histopathology. The clinical stage was IIIA (T3N0M0). Postoperative AFP was 99 ng/mL. The patient was started on adjuvant therapy with four cycles of cisplatin plus doxorubicin. The postoperative course was uneventful and the Kehr drain was removed two months later. The AFP after adjuvant chemotherapy was 7 ng/mL.

Case 2

**Figure 2:**
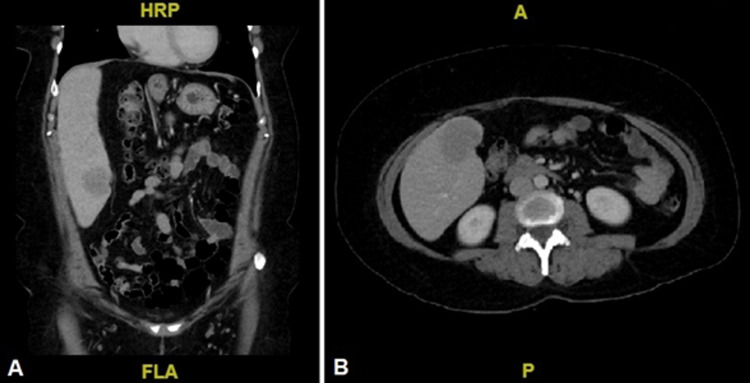
Abdominal CT scan, venous phase. Hypoattenuating hepatic lesion with lobulated margins located in Segment VI, compatible with disease recurrence. A: Coronal view. B: Axial view

A 45-year-old female with type 2 diabetes mellitus presents with a 12-month history of burning pain in the epigastrium. Family history was remarkable for HCC in her father and in four paternal cousins. The physical exam was unremarkable. Alpha-fetoprotein on admission was 27734 ng/mL. Serology for viral hepatitis A, B, and C was negative. Abdominal CT scan demonstrated a 7.2 cm hypodense lesion with discrete contrast uptake in liver Segments II and III. A left hemihepatectomy was performed, which revealed a pearly white tumor with lobulated edges located in Segments II, III, and IV. A single 9.5 cm FLC with clean surgical margins was confirmed by histopathology. The FLC was moderately differentiated with negative LVI and PNI and no lymph nodes were resected. The clinical stage was IIIA (T3N0M0). Postoperative AFP was 5 ng/mL. At the 39-month follow-up, AFP increased to 137 ng/mL without obvious lesions seen on imaging. Three months later, the AFP value tripled with no evidence of lesions in the positron emission tomography (PET) scan. At the 47-month follow-up, AFP rose to 1082 ng/mL. A repeat CT scan showed a hypodense lesion in Segment VI (Figure 2). The patient underwent hepatic bisegmentectomy of Segments V and VI. A 5 cm tumor was resected and recurrence of FLC was confirmed by histopathology. The patient was treated with nine cycles of capecitabine plus oxaliplatin. Alpha-fetoprotein at the end of adjuvant therapy decreased to 33 ng/mL.

Case 3

**Figure 3:**
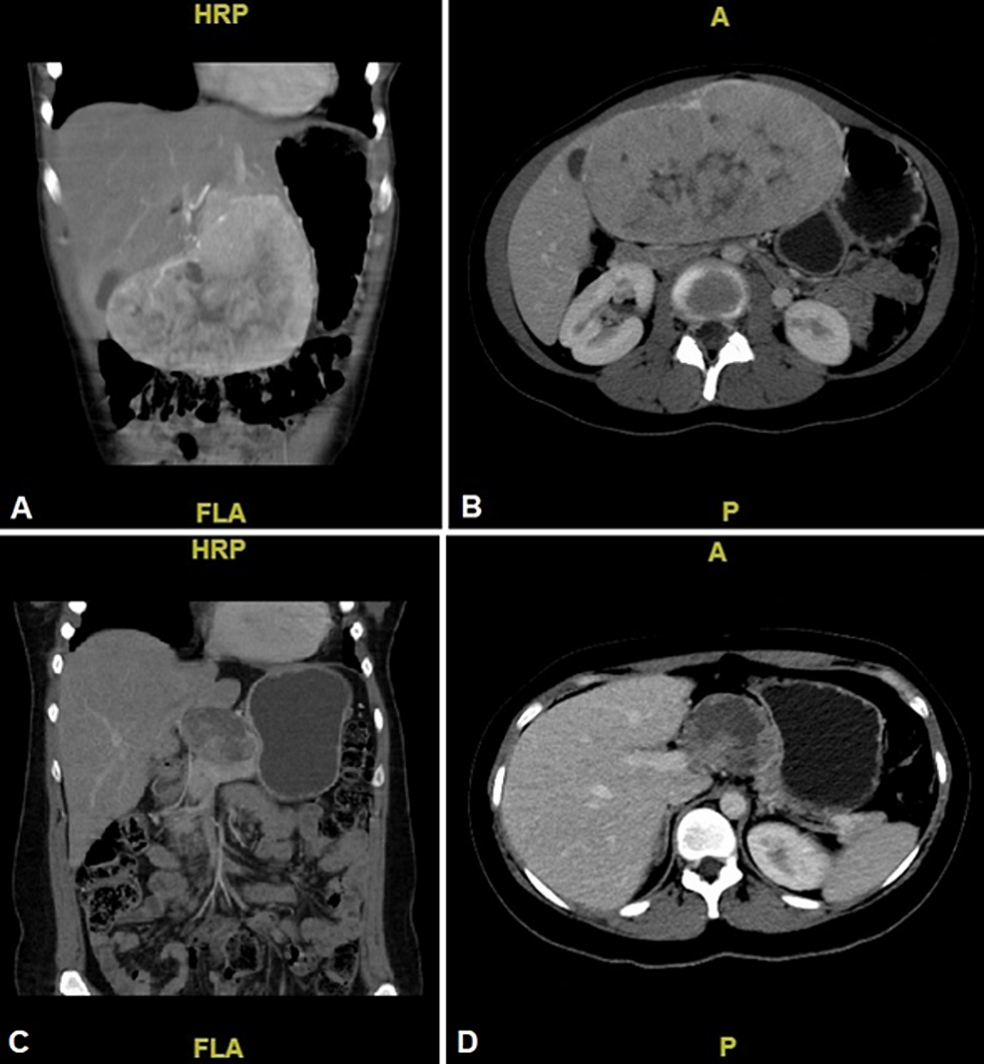
Abdominal CT scan, venous phase A and B: Coronal and axial views showing an extensive solid, heterogeneous, and hypervascular lesion in Segments II and III, with calcifications. C and D: Coronal and axial views of FLC in close contact with the head of the pancreas, compatible with disease recurrence. FLC: fibrolamellar carcinoma

A 24-year-old female with no significant past medical history presented with pain in the upper abdomen associated with nausea, vomiting, and a palpable mass in the epigastrium for the past 12 months. An abdominal magnetic resonance imaging (MRI) showed extensive "pedunculated" mass lesions in liver Segments II and III. The initial AFP was 1.54 ng/mL. The patient underwent hepatic bisegmentectomy where a multilobed tumor (14.0 cm) was found, with confirmatory histopathology for moderately differentiated FLC with negative surgical margins, LVI, and PNI. The clinical stage was IIIA (T3N0M0). Postoperative AFP was <1.3 ng/mL. At 13 months postoperatively, she presented with persistent epigastric pain. An abdominal CT scan revealed a 6 cm retroperitoneal mass lesion in close contact with the head of the pancreas (Figure 3). Alpha-fetoprotein remained unchanged. The patient then underwent hepatoduodenal ligament lymphadenectomy and will start systemic therapy.

Case 4

**Figure 4:**
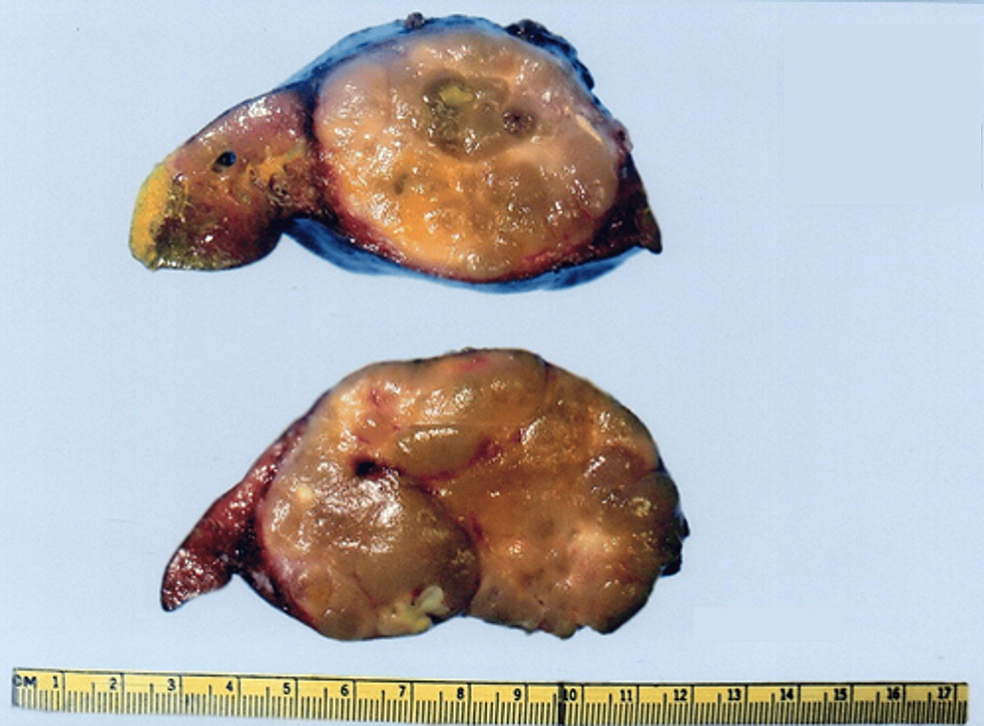
Surgical specimen from the left hemihepatectomy. A well-circumscribed tumor with a central grayish-white scar is evident.

A seven-year-old boy with a family history of grandparents with lung and brain cancer presented with a two-month history of epigastric pain. An abdominal CT scan showed a solid lesion with poorly defined borders and areas of necrosis located in liver Segments II and III. Initial AFP was 1.89 ng/mL. The patient had a left hemihepatectomy with gross findings of a pearly white, multilobed, and umbilicated tumor measuring 8.5 cm (Figure 4). The histopathology report confirmed moderately differentiated FLC partially infiltrating the hepatic capsule with no LVI or NPI. Clinical stage IIIA (T3N0M0). The postoperative AFP was 1.3 ng/mL. The patient was being followed with yearly ultrasound surveillance for nine years, with no signs of recurrence to this point.

Case 5

**Figure 5:**
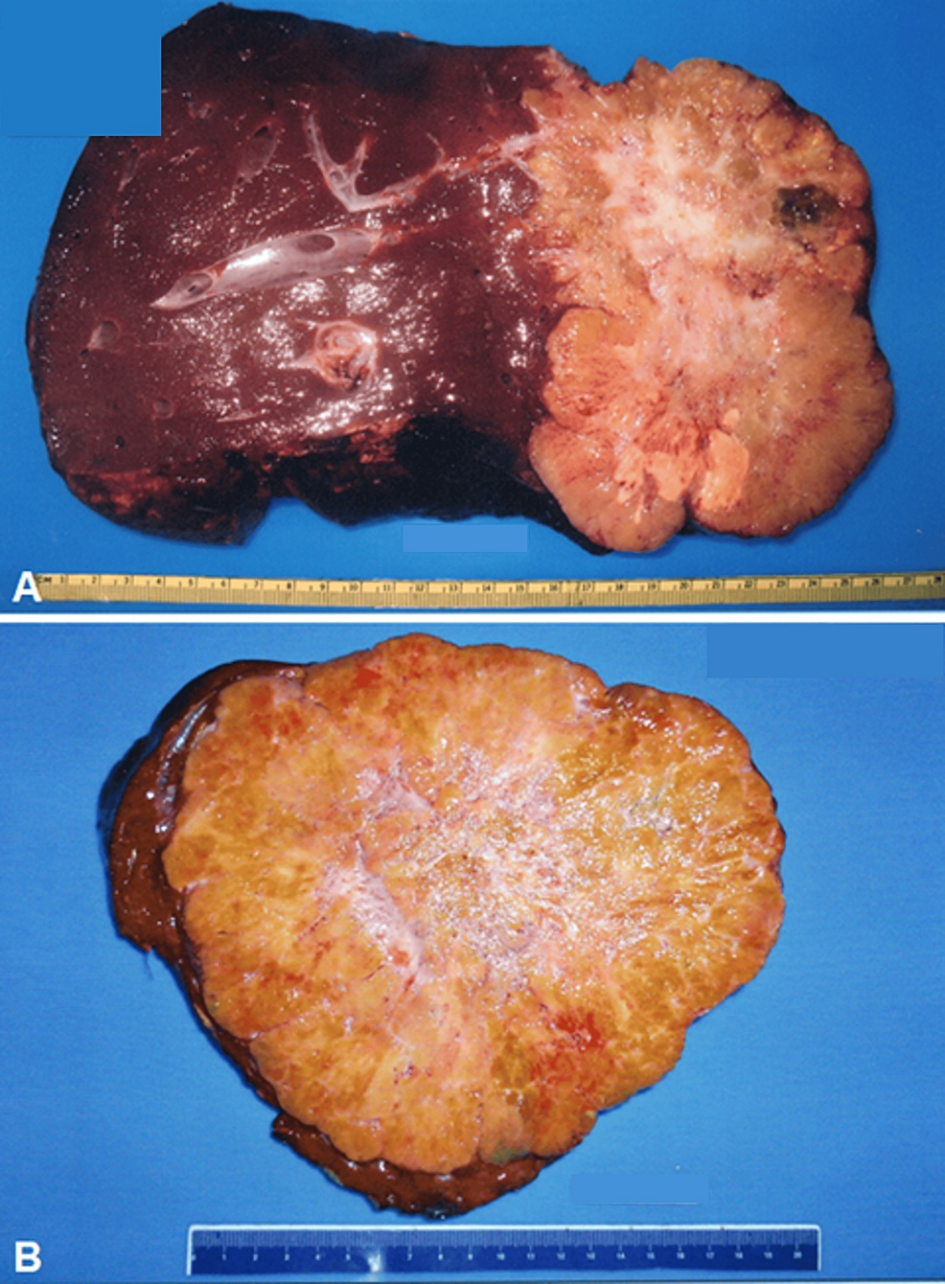
A. Surgical specimen from the right hemihepatectomy. A tumor with a multinodular surface and central scar is observed. B. Surgical specimen from the hepatic bisegmentectomy of Segments II and III. Recurrence of FLC with similar macroscopic characteristics to the primary tumor. FLC: fibrolamellar carcinoma

A 26-year-old male presented with a six-month history of abdominal pain and a palpable mass in the right upper quadrant. A solid, lobulated mass with heterogeneous contrast uptake and areas of necrosis was evidenced by abdominal CT in liver Segments V and VI. The initial AFP was 3.0 ng/mL. The patient had a right hemihepatectomy (Figure 5A). A 15.5 cm tumor adhered to the greater omentum and transverse mesocolon. Lymph nodes with metastatic appearance were resected in the hepatic hilum (groups 3, 8, and 9). The histopathology report demonstrated moderately differentiated FLC with clean surgical margins, negative LVI and PNI, and no nodal involvement. The clinical stage was IIIA (T3N0M0). The postoperative AFP was 3.15 ng/mL. The patient was then treated with five courses of adriamycin. At the 27-month follow-up, the abdominal ultrasonography showed a heterogeneous solid mass of 14 cm x 13 cm in the left lobe of the liver. The patient then underwent a palliative hepatic bisegmentectomy of liver Segments II and III since the tumor was fixed to the diaphragm (Figure 5B). The histopathology report confirmed FLC without vascular or parenchymal involvement.

## Discussion

Fibrolamellar carcinoma is an uncommon neoplasia worldwide and most data are obtained from case reports and registry-based studies [1,2]. Although some series have reported a prevalence as high as 5% [1], the results vary depending on the population and study design. A large retrospective study from 20 years of liver resection practice for HCC in Peru found that FLC represented around 1.5% of all cases [7], which is similar to what has been reported elsewhere [1]. We present a series of five patients with histopathological confirmation of FLC at the National Institute of Neoplastic Diseases in Peru from 2010 to 2021.

One of the most illustrative features of FLC is that affects young individuals. The largest series have reported a median age of diagnosis from 21 to 32 years [4,5,8]. In our series, the median age was 24 years old (range 7 to 45 years old). Although a sex predominance has also been described [4], other authors [1,5] and our series (male-to-female ratio of 1:1.5) describe similar proportions between both sexes.

The median time interval between onset of symptoms and diagnosis was six months (range 2-12 months). The most common symptoms were abdominal pain and palpable abdominal mass (Table 1). This is consistent with previous reports in which chronic gastrointestinal symptoms generally started 4 to 12 months before FLC diagnosis [2,9]. Patients are generally otherwise healthy with no prior liver disease [5], as shown in our series (median Charslon comorbidity index of zero). Normal laboratory liver profiles, the gross sample findings, and Ishak scores <1 allowed us to confidently state that there were no signs of chronic liver disease in our patients. In relation to this, less than 5% of FLC present with liver cirrhosis, in contrast to the classic presentation of HCC [8].

**Table 1:** Demographic, clinical, laboratory, and imaging features in our series ^a^Charlson comorbidity index calculated excluding the presence of a solid tumor AFP: alpha-fetoprotein; AST: aspartate aminotransferase; ALT: alanine aminotransferase; INR: international normalized ratio

Demographic features	
Age (years), median (range)	24 (7.0-45.0)
Sex, female, n (%)	3 (60.0)
Clinical features	
Time interval between onset of symptoms and diagnosis (months), median (range)	6 (2.0-12.0)
Symptoms, n (%)	
Abdominal pain	5 (100.0)
Palpable abdominal mass	3 (60.0)
Nausea/Vomiting	1 (20.0)
Charlson Comorbidity index^a^, median (range)	0 (0.0-1.0)
Prior known liver disease, n (%)	0 (0.0)
Laboratory features	
Preoperative AFP (ng/mL), median (range)	3.2 (1.5-27734.0)
AST/ALT ratio, median (range)	0.7 (0.5-1.5)
Albumin (g/L), mean ± SD	41.2 ± 2.6
INR, mean ± SD	1.1 ± 0.1
Imaging features	
Fibrous central scar (yes), n (%)	5 (100.0)
Microcalcifications (yes), n (%)	5 (100.0)
Areas of necrosis (yes), n (%)	2 (40.0)

With regards to laboratory features, serum levels of aspartate aminotransferase (AST), alanine aminotransferase (ALT), or ALP can be normal or mildly elevated in FLC [1]. Other markers studied include transcobalamin I, transcobalamin 2, vitamin B12 binding capacity, neurotensin, and Des-gamma carboxyprothrombin or PIVKA-II (protein induced by vitamin K absence/antagonist-II); however, additional evidence is needed to support their role as a tool for FLC diagnosis [1,3]. Alpha-fetoprotein is the most widely serum marker used and is usually within normal (<20 ng/mL) or slightly increased values, although 5 to 10% of patients may present with levels higher than 400 ng/mL [5]. In our series, AFP was highly variable, with values ​​between 1.54 and 27734 ng/mL (median of 3.2 ng/mL) at the time of diagnosis. Only two patients had AFP greater than 2000 ng/mL, and neither had extrahepatic involvement. Of note, an elevated AFP in FLC is more common in older patients with larger and poorly differentiated tumors [10]. Also, patients with higher levels of AFP have decreased overall survival (43 vs. 134 months) when compared to those with normal AFP levels with FLC [10].

Radiological evaluation for patients with FLC includes a CT scan or MRI. The usual CT scan findings include a well-demarcated, large, single, and heterogeneous mass with calcifications in the liver without imaging evidence of cirrhosis [6]. Tumors may also present with a central scar, areas of necrosis, and arterial enhancement [6]. All of our patients had a fibrous central scar and microcalcifications while only two (40%) had areas of necrosis (Table 1). Although MRI is also helpful for diagnostic guidance, only one patient had it due to its higher cost and lower availability. Along the same line, the MRI usually demonstrates a hypointense signal on T1-weighted imaging and hyperintense on T2-weighted imaging [6].

Macroscopically, the presence of a large, pale tumor with a central scar within a grossly healthy liver is classic [1,2], as seen in Figure 4 and Figure 5. The mean tumor size was 10.8 cm with a median of two segments involved (Table 2). Our results were similar to published data, with a median tumor size between 9 and 12 cm [5,8]. Other frequent characteristics are macroscopic vascular invasion and rupture of the hepatic capsule [1,2], both absent in our patients.

**Table 2:** Pathological features, treatment, and outcomes in our series

Pathological features	
Macroscopic	
Healthy liver appearance, n (%)	5 (100.0)
Tumor size (cm), mean ± SD	10.8 ± 3.8
Number of segments involved, median (range)	2 (2.0-3.0)
Microscopic	
Ishak score ≤1, n (%)	5 (100.0)
Differentiation (moderately differentiated), n (%)	5 (100.0)
Surgical margins (free), n (%)	5 (100.0)
Lymphovascular invasion (negative), n (%)	4 (80.0)
Perineural invasion (negative), n (%)	5 (100.0)
Treatment	
Initial surgery approach, n (%)	
Hemihepatectomy	4 (80.0)
Bisegmentectomy	1 (20.0)
Systemic adjuvant therapy, n (%)	3 (60.0)
Outcomes	
Discharge disposition (alive), n (%)	5 (100.0)
Follow-up (months), median (range)	36.1 (7.0-114.4)
Recurrence, n (%)	3 (60.0)

The tissue pathology remains the cornerstone for the diagnosis of FLC in addition to the clinical and radiological characteristics consistent with the usual presentation of the disease. Unfortunately, there are no specific tests for diagnostic confirmation. Immunohistochemistry is typically positive for HepPar1, pCEA, and glypican-3, however, this is also seen in HCC. Nonetheless, cytokeratin 7 and epithelial membrane seem to be specific for FLC [1,3]. Of note, a fusion gene (DNAJB1-PRKACA) between DNAJB1 (DnaJ/HSP40 homolog, subfamily B, member 1) and PRKACA (protein kinase, cAMP-dependent, catalytic, alpha) was initially thought to be specific for FLC but it has also been identified in pancreatic and biliary neoplasms [11].

**Figure 6:**
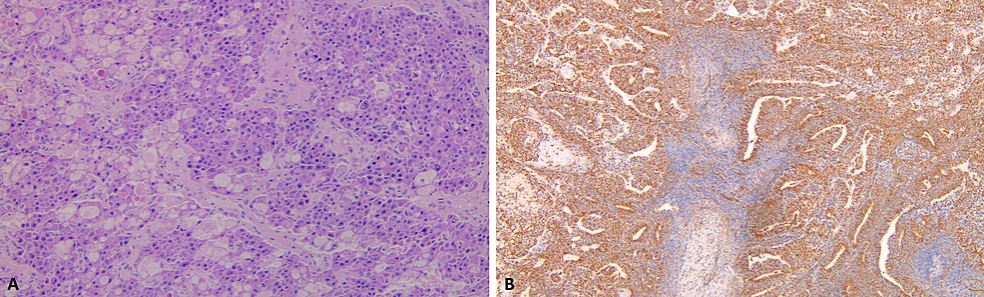
Histopathology showing large polygonal cells with eosinophilic cytoplasm (A, hematoxylin and eosin stain) embedded in lamellar bands of fibrosis (B, Hep Par 1 staining).

Microscopically, our patients had the classic large polygonal cells with eosinophilic cytoplasm embedded in lamellar bands of fibrosis (Figure 6) [1,2]. None of our patients had signs of fibrosis or chronic liver disease (Ishak score ≤1) as previously described in the literature [6,8]. Similarly, all of our patients had a moderately differentiated FLC, which is higher than what the largest series have reported (20-25%), however, the tumor grade remained unknown for more than 60% of the patients analyzed [4,5].

When the disease is localized (around 60% to 70% of FLC cases) surgery is the treatment of choice [3,5]. It has been reported that more than 70% of patients amenable to surgery require hemihepatectomy or extended hepatectomy [3,5]. All of our patients were surgical candidates and 80% underwent a hemihepatectomy. Along the same line, our FLC patients had negative surgical margins and no perineural invasion, and only one had lymphovascular invasion (Table 2). As expected, a tumor resection with free margins and sufficient liver mass to ensure future liver function is of vital importance for prognosis [2,3]. Likewise, vascular invasion and lymph node involvement have been associated with poorer outcomes after surgical treatment [2,4]. Unfortunately, FLC generally presents at advanced stages with regional lymph node involvement and metastases to the liver, lungs, and peritoneum [2,3]. As a result, surgery is not possible in most cases due to the advanced stage of the disease, requiring the use of chemotherapy. Three of our patients (60%) received systemic adjuvant therapy. The regimens were based on platinum, doxorubicin, and capecitabine. Regrettably, chemotherapy is ineffective for FLC and there are no standard recommendations for treatment [5]. Nonetheless, a phase II clinical trial with 5- fluorouracil (5-FU) and recombinant interferon α-2B (IFN-α-2B) has shown promising results [12].

Fibrolamellar carcinoma has a five-year survival rate of 35% to 55% [4,5,8] with a median overall survival of 39 months for any treatment, 74 months for surgical treatment, and 222 months for partial hepatectomy [8]. All of our patients were alive at discharge and on the date of the last contact (median follow-up of 36 months). A high recurrence rate has also been described, which can range from 50% to 100% in the first 3-5 years after resection [2,3]. Three of our patients (60%) had disease recurrence after the first year (at 13, 27 and 47 months). Recurrent disease can also undergo surgery, as in our three cases with recurrence, which confers better survival compared to nonoperative management, as demonstrated by the survival of 122 versus 37 months, respectively [13].

## Conclusions

While relatively rare, FLC is a unique tumor that predominantly affects younger individuals. Few studies examining risk factors, treatment modalities, and outcomes exist in the literature. We aim to add to the existing knowledge and provide a foundation for further research. Our findings support the existing literature around FLC by describing young, healthy patients who had better outcomes with surgery and high rates of recurrence. It is clear that more research needs to be done to better understand this neoplasia.
